# Generative AI-assisted learning and perceived academic performance in higher education: the indirect role of self-efficacy and the moderating role of AI literacy

**DOI:** 10.3389/fpsyg.2026.1949060

**Published:** 2026-10-06

**Authors:** Xiaotong Shen, Geumchul Um

**Affiliations:** 1 Department of Business, Gachon University, Seongnam, Republic of Korea; 2 Department of Business, College of Business, Gachon University, Seongnam, Republic of Korea

**Keywords:** AI, AI literacy, generative AI-assisted learning use, perceived academic performance, self-efficacy

## Abstract

**Introduction:**

Generative AI is increasingly used to support learning in higher education, yet the association between generative AI-assisted learning use and students’ perceived academic performance, as well as the conditions under which this association varies, remains unclear. Drawing on social cognitive theory and AI literacy research, this study tested whether generative AI-assisted learning use was indirectly associated with perceived academic performance through self-efficacy and whether AI literacy conditioned the association between generative AI-assisted learning use and self-efficacy.

**Methods:**

Cross-sectional questionnaire data were collected from 376 undergraduates at universities in China. An item-level latent interaction structural equation was estimated using a matched-pair product-indicator approach.

**Results:**

The model showed positive associations between generative AI-assisted learning use and self-efficacy (*β* = 0.207, *p* < 0.001) and between self-efficacy and perceived academic performance (*β* = 0.199, *p* < 0.001). The latent interaction between generative AI-assisted learning use and AI literacy was positive (*B* = 0.068, 95% bias-corrected bootstrap confidence interval [0.012, 0.123]), indicating that the association between generative AI-assisted learning use and self-efficacy was stronger at higher levels of AI literacy. The conditional indirect association was not statistically significant at low AI literacy but was positive and statistically significant at the mean and high levels. The index of moderated mediation was 0.011 (95% bias-corrected bootstrap confidence interval [0.002, 0.026]), indicating that the indirect association varied across levels of AI literacy.

**Discussion:**

These findings describe a theory-consistent conditional pattern of cross-sectional associations but do not establish temporal or causal ordering.

## 1 Introduction

As large language models such as ChatGPT become increasingly embedded in higher education, generative artificial intelligence has become an increasingly prominent resource that university students use to access knowledge, engage with learning materials, and complete academic tasks. Zawacki-Richter et al. (2019) noted that AI has been widely applied in higher education for learning support, educational administration, and technological innovation. Synthesizing evidence from a broad body of prior research, Crompton and Burke (2023) concluded that AI has rapidly expanded across higher education, with applications encompassing student assessment, predictive learning analytics, intelligent support systems, adaptive tutoring, and instructional management. Generative AI extends this educational landscape further. Kasneci et al. (2023) identified explanation generation, personalized feedback, and interactive tutoring as potential educational applications of large language models, while Yan et al. (2024) summarized their uses in feedback provision, content generation, assessment, and instructional support. Lo (2023) discussed both the potential instructional applications of ChatGPT and concerns regarding response accuracy, ethical practice, and assessment fairness. Dwivedi et al. (2023) and Cotton et al. (2024) likewise highlighted challenges involving information reliability, governance, policy regulation, and academic integrity. Against this background, scholarly attention has gradually moved beyond AI adoption toward examining how AI-supported learning is associated with educational experiences and outcomes and under what conditions these associations vary.

Higher education learning provides a broad context for examining generative AI-assisted learning. Students use generative AI not only for academic writing but also for explaining course content, summarizing literature, generating ideas, obtaining feedback, and managing learning tasks. Academic writing remains a salient application context because it combines knowledge comprehension, argument organization, drafting, and revision; however, the focal measures used in this study are not writing-specific. Köhler and Hartig’s (2024) ChatGPT Use Scale captures several learning activities that extend beyond writing, while the measures of self-efficacy and perceived academic performance assess general academic capability beliefs and subjective academic functioning. Accordingly, the present study is framed as an investigation of generative AI-assisted learning in higher education, with academic writing treated as an important illustrative context rather than the exclusive domain of inference.

The Chinese higher education context also requires specific consideration. Students may use domestic systems such as ERNIE Bot, Kimi, Doubao, and Tongyi Qianwen as well as global systems such as ChatGPT, Claude, and Gemini, but access, cost, language performance, culturally grounded content, and platform-specific content policies may differ across these systems. China’s Interim Measures for the Management of Generative Artificial Intelligence Services establish requirements concerning content governance, data protection, transparency, and service-provider responsibilities (Cyberspace Administration of China, 2023). Recent evidence further indicates that Chinese university students navigate global and domestic generative AI differently because of accessibility, linguistic, and cultural considerations (Xie et al., 2025). These contextual differences may be relevant to reported AI use and AI literacy; however, the present study did not measure platform-specific constraints and therefore does not test their separate associations.

Despite the progress made by previous studies, several unresolved issues continue to warrant further investigation. First, prior studies have mainly examined the opportunities, risks, student attitudes, and policy governance associated with generative AI. Chan (2023) introduced an institutional policy model for university AI implementation that integrates teaching practice, administrative governance, and operational management. Complementing this perspective, Chan and Hu (2023) reported that students simultaneously recognized both the educational opportunities and the potential drawbacks associated with generative AI. However, less is known about whether generative AI-assisted learning use is associated with students’ perceived academic performance through theory-guided psychological pathways. Related but conceptually distinct research has begun to examine conditional psychological associations in human–AI interaction. In a Chinese adult sample, Shen et al. (2026) reported a cross-sectional indirect statistical association between social anxiety and psychological resilience through AI chatbot dependence, with this association varying across levels of perceived social support. Although the constructs, population, and outcome domain differ from those examined in the present study, this evidence illustrates the value of considering both intervening constructs and boundary conditions in human–AI research. Second, most studies addressing technology adoption have concentrated on users’ judgments regarding a system’s practical value, the effort required for its use, and their willingness to embrace emerging technologies. Representative frameworks include Davis's (1989) Technology Acceptance Model and the Unified Theory of Acceptance and Use of Technology proposed by Venkatesh et al. (2003). Yet, in educational settings, use intention or frequency alone may not fully account for variation in students’ learning-related perceptions. Third, further explanation is needed regarding whether AI literacy conditions the association between generative AI-assisted learning use and learning-related perceptions. According to Long and Magerko (2020), AI literacy should be viewed as a broad capability consisting of conceptual knowledge about AI, appropriate practical use, critical judgment of AI-generated information, and effective engagement with intelligent technologies. Building on this perspective, Wang et al. (2023) measured AI literacy using four complementary dimensions, namely awareness, practical use, evaluative capability, and ethical responsibility.

To address these gaps, this study develops its research model based on social cognitive theory and self-efficacy theory. Bandura (1986) proposed that relationships between environmental resources and individual outcomes depend partly on individuals’ cognitive appraisals of tasks, capabilities, and environmental resources. Bandura (1997) further theorized self-efficacy as relevant to goal setting, effort, persistence, and performance. In academic settings, previous research has associated self-efficacy with learning strategies, cognitive engagement, motivation, and classroom performance (Pajares, 1996; Pintrich and De Groot, 1990). Accordingly, the present study treats self-efficacy as a theoretically proposed intervening variable in the association between generative AI-assisted learning use and perceived academic performance.

In the proposed framework, feedback, modeling, explanation, and task scaffolding provide a theoretical basis for expecting generative AI-assisted learning use to be positively associated with students’ self-efficacy. According to Bruning et al. (2013), confidence in academic writing should be viewed as a multidimensional capability encompassing idea development, mastery of writing conventions, and self-regulatory control during the writing process. Hattie and Timperley (2007) characterized feedback as information that helps learners recognize discrepancies between current performance and intended goals, while Wood et al. (1976) described scaffolding as external support for completing difficult tasks. These perspectives provide a theoretical rationale for expecting students who report more frequent generative AI-assisted learning use to also report stronger confidence in completing academic tasks; the present design tests this association rather than a causal change in confidence.

This study further proposes AI literacy as a contextual condition under which the association between generative AI-assisted learning use and self-efficacy may vary. Students with stronger AI literacy may be better able to formulate purposeful prompts, critically assess AI responses, and integrate AI-generated feedback into their learning practices. Students with relatively limited AI literacy may use AI frequently without reporting correspondingly stronger confidence in their own learning capabilities. Accordingly, this study examines whether generative AI-assisted learning use is associated with perceived academic performance indirectly through self-efficacy and whether AI literacy conditions the first-stage association in higher education. The model therefore represents a theory-guided conditional pattern of associations rather than an empirically established causal process.

## 2 Theoretical background and research hypotheses

### 2.1 Generative AI-assisted learning use in higher education

Generative AI-assisted learning use refers to students’ reported frequency of using generative AI tools to obtain explanations, summarize texts, generate ideas, receive feedback, revise expressions, organize written work, and manage learning tasks in higher education. In contrast to technology-acceptance research that focuses on adoption intention, the present study examines reported engagement with generative AI during authentic academic activities. The construct therefore represents a broad higher education measure of the frequency of generative AI-assisted learning use rather than a writing-specific behavior or a measure of the pedagogical quality of AI use.

Academic writing in higher education is a cognitively demanding learning activity. It often involves topic development, literature comprehension, idea generation, argument organization, text drafting, revision, and final integration. Students are required not only to understand course knowledge but also to transform that knowledge into academically appropriate texts with clear structure, coherent logic, and scholarly conventions. Köhler and Hartig (2024) developed and validated the ChatGPT Use Scale, showing that university students use ChatGPT for practical learning tasks such as receiving text feedback, explaining course content, generating writing outlines, summarizing texts, obtaining topic overviews, developing ideas, and managing learning tasks. These uses are closely aligned with the academic writing process. Barrett and Pack (2023) described the use of generative AI across idea generation, drafting, revision, and feedback interpretation. Using a mixed-methods intervention design, Mahapatra (2024) reported higher academic writing performance among undergraduate ESL learners who participated in ChatGPT-supported formative feedback activities. Collectively, these studies situate generative AI across multiple stages of academic learning and writing, but they do not establish that frequency of AI use alone is educationally beneficial.

Nevertheless, frequency of generative AI-assisted learning use should not be equated with educational value. The same reported frequency may reflect substantially different practices, ranging from reflective feedback engagement and iterative revision to direct answer generation or copying. Such differences may be associated with different levels of task engagement, perceived control, overreliance, and academic-integrity risk (Cotton et al., 2024; Dwivedi et al., 2023). Drawing on social cognitive theory, this study conceptualizes generative AI-assisted learning use as a reported external learning resource and tests its association with perceived academic performance, including an indirect statistical association through self-efficacy.

### 2.2 Generative AI-assisted learning and self-efficacy

From the perspective of social cognitive theory, self-efficacy reflects learners’ confidence in their capacity to successfully carry out task-related behaviors and achieve intended learning goals (Bandura, 1986, 1997). In learning contexts, self-efficacy has been associated with students’ goal setting, effort, persistence, learning strategy use, and performance outcomes. Pajares (1996) identified self-efficacy as an important construct for explaining students’ academic motivation, self-regulation, and academic performance. Pintrich and De Groot (1990) found that self-efficacy is closely associated with learning strategy use, cognitive engagement, and classroom performance. Zimmerman et al. (1992) further reported an association between self-efficacy and academic attainment that was statistically linked to personal goal setting.

Self-efficacy is particularly important in academic writing. Academic writing requires students to comprehend literature, generate ideas, organize arguments, express ideas clearly, and revise their texts through multiple rounds. Pajares and Valiante (2006) described consistent relationships between writing self-efficacy, writing motivation, persistence, and writing performance. Bruning et al. (2013) further conceptualized writing self-efficacy as a multidimensional construct involving idea generation, writing conventions, and self-regulation. Thus, whether students believe they can complete academic writing tasks constitutes an important psychological foundation for their learning processes and performance evaluations.

The proposed model expects a positive association between generative AI-assisted learning use and students’ self-efficacy, with feedback, modeling, explanation, and task decomposition providing the theoretical rationale for this expectation. First, generative AI can provide immediate feedback. When students write course papers, summarize literature, or revise paragraphs, they can quickly receive suggestions on argument logic, structural organization, expression accuracy, and content improvement. According to Hattie and Timperley (2007), well-designed feedback enables learners to recognize discrepancies between their existing level of performance and their intended learning objectives while providing guidance for subsequent improvement. Consistent with this perspective, Nicol and Macfarlane-Dick (2006) argued that constructive feedback plays an important role in fostering students’ capacity for self-regulation. In generative AI-assisted academic writing, AI feedback may therefore help students recognize problems, understand revision pathways, and develop the belief that they can improve and complete the task.

Second, generative AI can provide examples and explanations. For students with limited writing experience, building a paper structure, developing arguments, and integrating literature may be difficult. AI-generated examples, explanations, and structural suggestions can function as task demonstrations. Bandura (1997) identified vicarious experience as an important source of self-efficacy, suggesting that individuals may strengthen their confidence in completing similar tasks by observing examples or referring to how tasks are performed. Third, generative AI can break complex writing tasks into more specific and manageable steps. Wood et al.’s (1976) account of scaffolding suggests that external support can help learners accomplish tasks they may not yet be able to complete independently, while gradually increasing their sense of task mastery. Thus, the availability of writing plans, step-by-step suggestions, and revision checklists provides a theoretical basis for expecting generative AI-assisted learning use to be positively associated with students’ perceived control over academic tasks.

A higher frequency of generative AI-assisted learning use should not be interpreted as evidence that AI use improved self-efficacy. The measure used in this study does not distinguish reflective feedback engagement from direct answer generation and therefore does not capture the quality of students’ AI use practices. The present study tests whether students reporting more frequent generative AI-assisted learning use also report higher self-efficacy, while recognizing that unmeasured differences in use quality may account for heterogeneity in this association. Therefore, this study proposes:

*H1*: Generative AI-assisted learning use is positively associated with students’ self-efficacy.

### 2.3 Self-efficacy and perceived academic performance

Perceived academic performance refers to students’ subjective evaluation of whether they meet academic requirements, complete learning tasks, and fulfill course-related responsibilities. In cross-sectional studies, perceived academic performance represents individuals’ overall appraisal of their learning progress together with the outcomes they believe they have achieved. To assess this construct, the scale developed by Verner-Filion and Vallerand (2016) was adopted, as it evaluates whether respondents perceive themselves as meeting course expectations, completing required learning tasks, and fulfilling the academic responsibilities associated with their coursework. It is therefore suitable for measuring students’ subjective assessment of their own academic performance.

Self-efficacy is expected to be positively associated with perceived academic performance. Bandura (1997) theorized self-efficacy as relevant to effort, persistence, and emotional responses during task performance. Pajares (1996) described relationships between academic self-efficacy, motivation, and learning outcomes. Pintrich and De Groot (1990) reported significant associations among self-efficacy, cognitive engagement, learning strategy use, and classroom performance, while Zimmerman et al. (1992) reported an association between self-efficacy and academic attainment involving personal goal setting. Taken together, these studies provide a theoretical basis for expecting students with higher self-efficacy to report more favorable evaluations of academic performance.

In higher education academic writing, students with higher self-efficacy are more likely to believe that they can understand course materials, organize arguments, complete writing tasks, and meet course requirements. Such capability beliefs may be reflected in how students interpret and report their learning processes and academic functioning. When learners are confident in their ability to accomplish academic writing tasks, they tend to assess their course performance, task completion, and fulfillment of academic responsibilities more favorably. Conversely, students with lower self-efficacy may underestimate their own performance even after completing required tasks because they lack confidence in their academic abilities. Therefore, this study proposes:

*H2*: Students with stronger self-efficacy are expected to report higher levels of perceived academic performance.

### 2.4 Self-efficacy as an intervening variable in the indirect association

Building on the preceding arguments, this study proposes that generative AI-assisted learning use is positively and indirectly associated with perceived academic performance through self-efficacy. This indirect association is theoretically grounded in social cognitive theory. Bandura’s (1986, 1997) framework provides a rationale for examining cognitive appraisals between reported environmental resources and individual outcomes. Within the proposed model, generative AI-assisted learning use is conceptualized as an external learning resource, and self-efficacy is examined as an intervening variable in its statistical association with perceived academic performance.

Specifically, students who report using generative AI more frequently for suggestions, explanations, structural prompts, and revision feedback may also report stronger perceived control over academic tasks. If this perceived control is reflected in higher self-efficacy, generative AI-assisted learning use may show an indirect statistical association with perceived academic performance. Thus, the empirical question is whether self-efficacy accounts for part of the covariance between generative AI-assisted learning use and perceived academic performance, without implying temporal or causal mediation. Therefore, this study proposes:

*H3*: Generative AI-assisted learning use is positively and indirectly associated with perceived academic performance through self-efficacy.

### 2.5 AI literacy as a boundary condition

AI literacy is commonly regarded as a broad set of competencies that enable individuals to comprehend AI technologies, apply them appropriately, critically assess their outputs, and communicate effectively with intelligent systems. Consistent with this perspective, Long and Magerko (2020) described AI literacy as a multidimensional capability extending well beyond isolated technical skills. This framework emphasizes foundational knowledge of AI principles, the ability to identify appropriate real-world uses of AI, critical judgment regarding both the capabilities and constraints of intelligent technologies, and effective collaboration with AI-enabled systems. Ng et al. (2021) further suggested that AI literacy should include cognitive, operational, evaluative, and ethical dimensions. Laupichler et al. (2022), in a scoping review of AI literacy in higher and adult education, indicated that AI literacy research is moving beyond general AI knowledge toward competency development for different learners and application contexts. Wang et al. (2023) operationalized AI literacy through four dimensions: awareness, use, evaluation, and ethics. Southworth et al. (2023) further emphasized that AI literacy in universities should not be treated as isolated technical training, but should be integrated into interdisciplinary curricula so that students can use AI critically and responsibly in authentic learning and professional tasks.

In higher education learning, AI literacy may condition the strength of the association between reported generative AI-assisted learning use and self-efficacy. Individuals with advanced AI literacy are generally better equipped to judge when AI assistance should be employed, formulate prompts aligned with specific learning objectives, and identify inaccurate, biased, or academically inappropriate AI responses. Before incorporating AI-generated suggestions into their own work, these learners typically evaluate, compare, and refine the information rather than accepting it without reflection. In contrast, students with lower AI literacy may report frequent AI use without reporting correspondingly higher self-efficacy, potentially because of less purposeful prompting, limited evaluation of AI outputs, or uncritical reliance on those outputs.

In educational settings, AI literacy is closely associated with students’ ability to apply generative AI in an appropriate, ethical, and academically responsible manner. Chan (2023) proposed that higher education institutions should establish an institution-wide policy system for AI implementation that integrates teaching practice, organizational governance, and day-to-day operational management. UNESCO (2023) also emphasized that the use of generative AI in education and research should be guided by human agency, transparency, safety, and ethical responsibility. Accordingly, AI literacy extends beyond technical proficiency in using AI tools and encompasses the broader competence to evaluate, apply, and manage AI technologies in a reflective, responsible, and effective manner. Drawing on social cognitive theory, learners’ appraisal and use of external resources provide a theoretical rationale for expecting the association between generative AI-assisted learning use and self-efficacy to vary across levels of AI literacy. Following the preceding theoretical arguments, it is expected that students with stronger AI literacy will be better equipped to identify appropriate situations for AI-supported learning, construct effective task-specific prompts, critically evaluate the accuracy and usefulness of AI responses, and integrate AI-generated feedback into their own understanding and subsequent revision processes. Under conditions of higher AI literacy, the positive association between generative AI-assisted learning use and self-efficacy is expected to be stronger. By comparison, individuals with relatively limited AI literacy may find it difficult to critically assess AI-generated responses, and they may rely on such outputs without sufficient verification or integrate them into their work without meaningful revision.

Under conditions of relatively limited AI literacy, the corresponding positive association is expected to be weaker. Therefore, this study proposes:

*H4*: AI literacy moderates the positive association between generative AI-assisted learning use and self-efficacy, such that the association is stronger at higher levels of AI literacy.

### 2.6 AI literacy and the conditional indirect association

Integrating the preceding arguments, this study further proposes a conditional indirect association. Preacher et al. (2007) showed that an indirect association may vary when one component association is conditional on a moderator. Building on this perspective, Hayes (2022) described such analytical frameworks as conditional process models and recommended evaluating conditional indirect associations using bootstrap confidence intervals and the index of moderated mediation.

Within the proposed model, the positive association between generative AI-assisted learning use and self-efficacy is expected to vary across levels of AI literacy. Because this represents the first-stage association in the theory-guided indirect model, the indirect association between generative AI-assisted learning use and perceived academic performance through self-efficacy is also expected to vary across levels of AI literacy. Specifically, the positive indirect association is expected to be stronger at higher AI literacy and weaker at lower AI literacy. This formulation describes heterogeneity in cross-sectional statistical associations and does not establish a temporal transformation process. Therefore, this study proposes:

*H5*: The positive indirect association between generative AI-assisted learning use and perceived academic performance through self-efficacy is stronger at higher levels of AI literacy. The proposed research model is shown in Figure 1.

**Figure 1:**
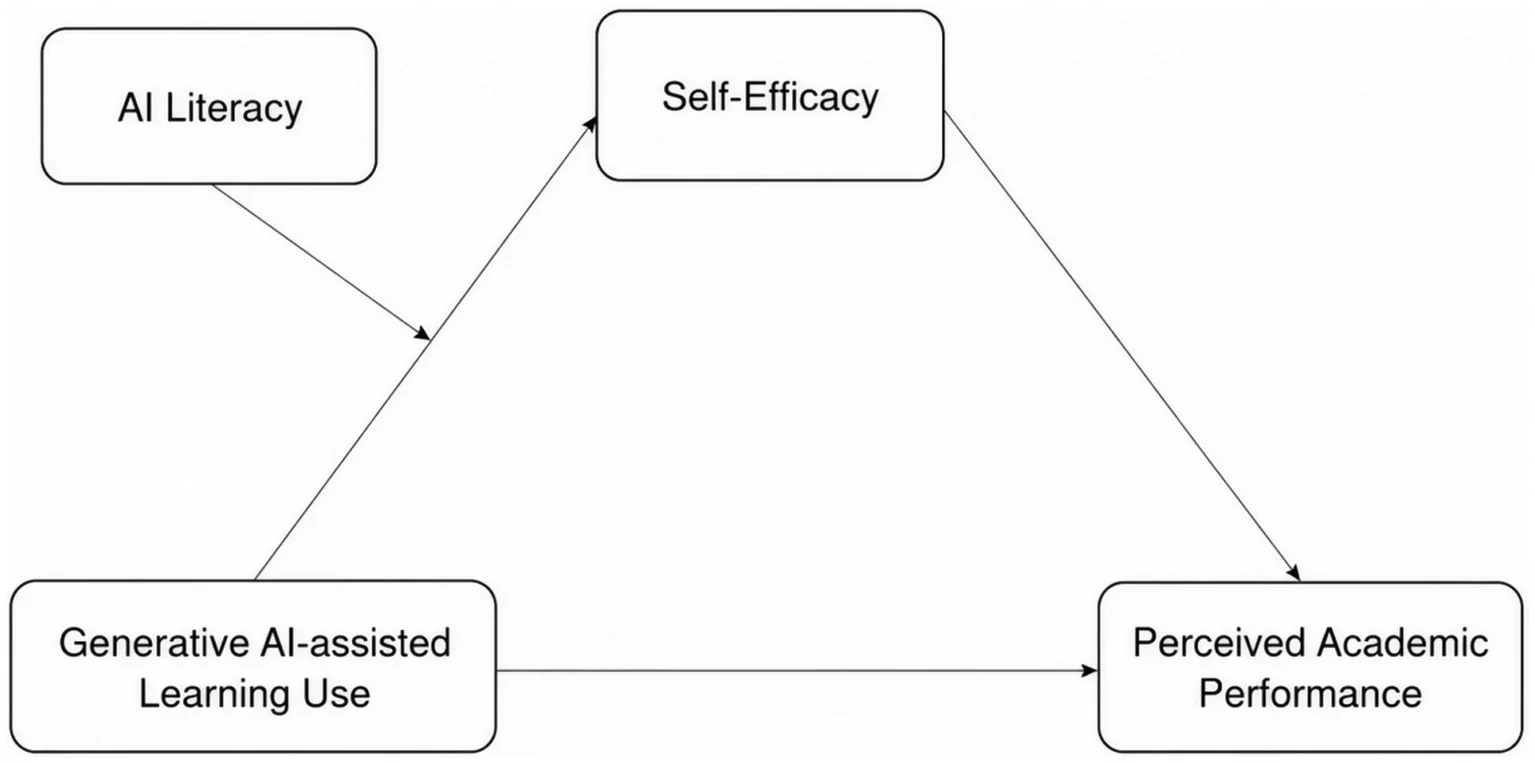
Research model.

## 3 Methodology

### 3.1 Participants and procedure

A cross-sectional questionnaire survey was conducted to investigate the associations among generative AI-assisted learning use, self-efficacy, AI literacy, and perceived academic performance in higher education learning contexts. Because the study examines students’ reported use of generative AI during authentic higher education learning activities and tests a theory-guided conditional pattern of associations, a questionnaire design was suitable for obtaining data from a sufficiently large sample; however, this design cannot establish temporal ordering or causality.

The target participants were undergraduate students enrolled in Chinese universities. This group was selected for three main reasons. First, undergraduate students are among the key users of generative AI-assisted learning, particularly in course papers, literature reading, text revision, and academic writing. Second, higher education learning involves substantial cognitive and self-regulatory demands, providing a relevant context for testing whether self-efficacy statistically links generative AI-assisted learning use with perceived academic performance. Third, undergraduate students vary in their levels of AI literacy, which provides an appropriate empirical setting for testing the boundary role of AI literacy.

The questionnaire was distributed through the Wenjuanxing online survey platform. Prior to answering the questionnaire, all respondents received an informed consent statement describing the objectives of the research, procedures for data handling, anonymity protection, voluntary involvement, and their freedom to discontinue participation at any stage without penalty. Only participants who confirmed informed consent proceeded to the questionnaire. To ensure that the sample matched the research context, screening questions were included. Participants were required to be currently enrolled undergraduate students and to have used generative AI tools during the previous semester to support course learning, academic writing, literature summarization, paper conceptualization, text revision, or assignment completion. In this study, generative AI tools refer to systems such as ChatGPT, Claude, Gemini, Kimi, ERNIE Bot, Doubao, and Tongyi Qianwen, which can generate text, explain knowledge, summarize materials, provide feedback, or assist with writing. Although respondents could indicate the generative AI tools they had used, the survey did not obtain separate frequency or quality scores for each platform; consequently, the analyses cannot distinguish associations attributable to domestic versus global systems or to particular platform policies. An attention-check item was embedded in the questionnaire. Of the 401 responses, 13 failed the attention check and 12 showed invariant, near-invariant, or alternating response patterns across the focal scales; no case was excluded solely because of completion time. After these 25 cases were removed, 376 valid cases remained for analysis (93.77%).

### 3.2 Measurement instruments

Each latent construct was assessed using well-established measurement instruments reported in previous studies. Minor wording modifications were introduced to ensure consistency with the broader higher education learning context. Generative AI-assisted learning use was captured with a seven-category frequency scale anchored from 1 (“never”) to 7 (“very frequently”). By comparison, self-efficacy, AI literacy, and perceived academic performance were measured with seven-point agreement scales. Responses closer to the upper end indicated stronger endorsement of each item, whereas lower scores represented greater disagreement (anchors: 1 = “strongly disagree”; 7 = “strongly agree”).

The construct of generative AI-assisted learning use was operationalized with the ChatGPT Use Scale introduced by Köhler and Hartig (2024). The scale includes 10 items related to learning tasks, covering text feedback, explanation of course content, generation of writing outlines, text summarization, idea generation, and learning-management suggestions. Considering that university students in China typically rely on multiple AI-based applications instead of a single platform, the original wording of the instrument was revised by substituting the term “ChatGPT” with the broader expression “generative AI tools.” Accordingly, the item set represents reported frequency across several academic activities and was modeled as a latent construct in the hypothesis tests. It does not measure the pedagogical quality of use, the accuracy of AI-generated outputs, reflective engagement with feedback, direct answer copying, or the degree of cognitive offloading.

Self-efficacy was operationalized using the Self-Efficacy for Learning and Performance dimension of the Motivated Strategies for Learning Questionnaire (MSLQ) proposed by Pintrich et al. (1991). This instrument comprises eight items assessing respondents’ confidence in course learning, mastery of academic tasks, and perceived academic capability.

Participants’ AI literacy was measured with the Artificial Intelligence Literacy instrument introduced by Wang et al. (2023). The scale consists of 12 items covering four dimensions: awareness, use, evaluation, and ethics. AI literacy was modeled as a latent construct represented by its 12 item indicators, and its interaction with generative AI-assisted learning use was estimated at the latent level rather than from a composite mean score.

Perceived academic performance was assessed using the measurement instrument proposed by Verner-Filion and Vallerand (2016). The scale includes five items and evaluates students’ subjective perceptions of whether they meet academic requirements, complete learning tasks, and fulfill course-related responsibilities. Perceived academic performance represents students’ subjective appraisal of academic functioning rather than objective achievement. It should not be interpreted as course grades, externally assessed writing quality, verified knowledge acquisition, or instructor-rated performance. Additionally, demographic and background information, including gender, age, grade level, and experience with generative AI, was collected to characterize the sample and support auxiliary analyses (see Appendix A).

### 3.3 Data analysis

IBM SPSS Statistics 26.0 was used for data screening, descriptive statistics, Pearson correlations, and Cronbach’s α. IBM SPSS Amos 26.0 was used to estimate the confirmatory factor models, the unmeasured latent method factor model, the baseline structural model, and the item-level product-indicator interaction model by maximum likelihood. Model adequacy was evaluated by considering several goodness-of-fit statistics, including χ^2^/*df*, CFI, TLI, RMSEA, and SRMR (Hu and Bentler, 1999; Kline, 2016). Convergent quality was judged according to standardized factor loadings together with composite reliability (CR) and average variance extracted (AVE). To determine whether the constructs were empirically distinguishable, the Fornell–Larcker approach and the heterotrait-monotrait (HTMT) index were jointly considered (Fornell and Larcker, 1981; Henseler et al., 2015).

Potential same-source method variance was addressed through both procedural and statistical approaches. Procedurally, the survey used anonymity assurances, modular presentation, an attention-check item, and separation of construct sections. Statistically, the primary assessment used an unmeasured latent method factor model in which every observed item loaded on its theorized trait factor and on a common method factor that was specified as orthogonal to the trait factors (Podsakoff et al., 2003). The fit of this model was compared with the four-factor trait-only measurement model, and the average squared standardized method loading was examined. For transparency, the item with the largest standardized method loading was identified and reported, while the method factor was kept orthogonal to the four trait factors. Harman’s single-factor result and full-collinearity VIFs were not treated as evidence that common method variance was absent. Because no *post hoc* procedure can eliminate same-source bias, the findings were interpreted cautiously.

After the measurement model was evaluated, the latent interaction was estimated using an unconstrained matched-pair product-indicator approach with double-mean centering (Marsh et al., 2004). The 10 indicators of generative AI-assisted learning use were paired with 10 AI literacy indicators having the most similar standardized factor loadings; the two remaining AI literacy indicators were not used to form products. Both constituent indicators were mean-centered before multiplication, and the resulting product indicators were centered again. The item-level model simultaneously regressed self-efficacy on generative AI-assisted learning use, AI literacy, and their latent interaction, and regressed perceived academic performance on generative AI-assisted learning use and self-efficacy. Parameters were estimated by maximum likelihood. Bias-corrected bootstrap confidence intervals based on 5,000 resamples were used for the latent interaction, simple slopes, indirect associations, and the index of moderated mediation. Because all focal variables were measured concurrently, all paths and indirect estimates were interpreted as statistical associations rather than causal effects.

## 4 Results

### 4.1 Data screening and sample characteristics

Overall, 401 survey responses were obtained during data collection. The dataset was screened using the criteria described in Section 3.1. Thirteen responses failed the attention check, and 12 showed invariant, near-invariant, or alternating response patterns across the focal scales; no case was excluded solely because of completion time. After these 25 cases were removed, 376 usable cases remained (93.77%). The retained dataset contained no missing values for any of the 35 focal construct indicators, and all item responses fell within the intended 1–7 range; therefore, no missing-value imputation or out-of-range recoding was required. All valid respondents were undergraduate students in Chinese universities who had used generative AI tools during the previous semester to support course learning, academic writing, literature summarization, paper conceptualization, text revision, or assignment completion.

Participant characteristics are summarized in Table 1. The final analytical sample consisted of 376 respondents, including 170 men (45.21%) and 206 women (54.79%). Participants ranged in age from 18 to 25 years, with a mean of 20.34 years (SD = 1.74). Regarding grade level, 75 respondents were freshmen (19.95%), 137 were sophomores (36.44%), 103 were juniors (27.39%), and 61 were seniors (16.22%). In terms of generative AI use experience, 45 students had used generative AI for less than 1 month (11.97%), 35 had used it for 1 to 3 months (9.31%), 95 had used it for 4 to 6 months (25.27%), 116 had used it for 7 to 12 months (30.85%), and 85 had used it for more than 1 year (22.61%).

**Table 1:** Participant characteristics.

Variable	Category	Frequency	Percentage
Gender	Male	170	45.21%
	Female	206	54.79%
Grade	Freshman	75	19.95%
	Sophomore	137	36.44%
	Junior	103	27.39%
	Senior	61	16.22%
Generative AI use experience	Less than 1 month	45	11.97%
	1–3 months	35	9.31%
	4–6 months	95	25.27%
	7–12 months	116	30.85%
	More than 1 year	85	22.61%

*N* = 376. Mean age = 20.34, SD = 1.74, range = 18–25.

### 4.2 Measurement model fit and potential common method effects

The hypothesized four-factor trait-only measurement model showed good fit (χ^2^ = 601.561, df = 554, χ^2^/df = 1.086, CFI = 0.996, TLI = 0.995, RMSEA = 0.015, SRMR = 0.034). In contrast, the single-factor model fit the data poorly (χ^2^ = 7062.843, df = 560, χ^2^/df = 12.612, CFI = 0.407, TLI = 0.370, RMSEA = 0.176, SRMR = 0.258), indicating that the 35 focal indicators were not adequately represented by a single latent factor.

Because all focal variables were obtained from the same respondents at one time point, an unmeasured latent method factor model was subsequently estimated as a sensitivity assessment. As reported in Table 2, adding an orthogonal common method factor improved model fit (χ^2^ = 529.441, df = 519, χ^2^/df = 1.020, CFI = 0.999, TLI = 0.999, RMSEA = 0.007, SRMR = 0.030), Δχ^2^(35) = 72.120, *p* < 0.001. The mean squared standardized method loading was 0.061 (approximately 6.1% on average); however, AIL9 had a standardized method loading of 0.712 (squared loading = 0.508), indicating that method-related covariance was not uniform across items. These findings indicate detectable and heterogeneous method-related covariance. The common method factor analysis should therefore be interpreted as a sensitivity assessment rather than as evidence that common method variance was absent or negligible. Common method variance remains a limitation, and the structural estimates are interpreted as conditional cross-sectional associations.

**Table 2:** Fit indices for the measurement and common method factor models.

Model	χ^2^	df	χ^2^/df	CFI	TLI	RMSEA	SRMR
Four-factor trait model	601.561	554	1.086	0.996	0.995	0.015	0.034
Single-factor model	7062.843	560	12.612	0.407	0.370	0.176	0.258
Four-factor + orthogonal common method factor model	529.441	519	1.020	0.999	0.999	0.007	0.030

*N* = 376. The common method factor was specified as orthogonal to the four trait factors. Relative to the four-factor measurement model, adding the common method factor produced Δχ^2^(35) = 72.120, *p* < 0.001. The mean squared standardized method loading was 0.061; AIL9 had the largest standardized method loading (0.712). CFI, comparative fit index; TLI, Tucker-Lewis index; RMSEA, root mean square error of approximation; SRMR, standardized root mean square residual.

### 4.3 Construct reliability and validity

Beyond the global model-fit results reported in Table 2, reliability and convergent validity were evaluated at the construct level. As reported in Table 3, Panel A, the standardized factor loadings ranged from 0.720 to 0.892, and all loadings exceeded the recommended minimum criterion of 0.50. Cronbach’s α coefficients ranged from 0.883 to 0.967, composite reliability values ranged from 0.883 to 0.967, and average variance extracted values ranged from 0.602 to 0.713. These results provide acceptable evidence of indicator reliability, internal consistency, and convergent validity for all four latent constructs.

**Table 3:** Descriptive statistics, reliability, correlations, and construct validity.

Panel A. Reliability, convergent validity, and descriptive statistics						
Construct	Loading range	α	CR	AVE	*M*	SD
1. GAIU	0.782–0.868	0.957	0.957	0.688	4.026	1.711
2. SE	0.787–0.847	0.946	0.946	0.685	4.357	1.786
3. AIL	0.789–0.892	0.967	0.967	0.713	4.324	1.739
4. PAP	0.720–0.813	0.883	0.883	0.602	4.259	1.704

Panel B. Correlations, square roots of AVE, and HTMT ratios				
Construct	1	2	3	4
1. GAIU	**0.830**	0.221	0.056	0.155
2. SE	0.210***	**0.828**	0.199	0.220
3. AIL	0.043	0.190***	**0.844**	0.085
4. PAP	0.141**	0.201***	0.073	**0.776**

Bold values on the diagonal in Panel B are the square roots of AVE; values below the diagonal are Pearson correlations; values above the diagonal are HTMT ratios. Means and standard deviations are based on averaged scale scores. GAIU, generative AI-assisted learning use; SE, self-efficacy; AIL, AI literacy; PAP, perceived academic performance; CR, composite reliability; AVE, average variance extracted. **p < 0.01. ***p < 0.001. Bold values on the diagonal in Panel B are the square roots of AVE; values below the diagonal are Pearson correlations; values above the diagonal are HTMT ratios.

Construct distinctiveness was evaluated using both the Fornell–Larcker criterion and the heterotrait-monotrait ratio. As shown in Table 3, Panel B, the square root of AVE for each construct exceeded its corresponding inter-construct correlations. The HTMT ratios reported above the diagonal ranged from 0.056 to 0.221 and were below the recommended threshold of 0.85. Taken together, these results provide acceptable evidence that generative AI-assisted learning use, self-efficacy, AI literacy, and perceived academic performance were empirically distinguishable.

### 4.4 Descriptive statistics and correlations

Descriptive statistics and zero-order Pearson correlations are reported in Table 3, Panels A and B. The mean scores of the four focal constructs ranged from 4.026 to 4.357 on the seven-point scales, with standard deviations ranging from 1.704 to 1.786. Generative AI-assisted learning use was positively correlated with self-efficacy (*r* = 0.210, *p* < 0.001) and perceived academic performance (*r* = 0.141, *p* < 0.01). Self-efficacy was also positively correlated with AI literacy (*r* = 0.190, *p* < 0.001) and perceived academic performance (*r* = 0.201, *p* < 0.001). By contrast, the correlations between generative AI-assisted learning use and AI literacy (*r* = 0.043, *p* > 0.05) and between AI literacy and perceived academic performance (*r* = 0.073, *p* > 0.05) were not statistically significant. These zero-order correlations describe bivariate associations only and do not test the latent interaction or the proposed conditional indirect association.

### 4.5 Baseline latent structural model and indirect association

After confirmation of the measurement model, a baseline latent structural model without the product-indicator interaction was estimated. The model included associations from generative AI-assisted learning use and AI literacy to self-efficacy and from generative AI-assisted learning use and self-efficacy to perceived academic performance. The model showed good fit: χ^2^ = 602.176, df = 555, χ^2^/df = 1.085, CFI = 0.996, TLI = 0.995, RMSEA = 0.015, and SRMR = 0.035.

As reported in Table 4, Panel A, generative AI-assisted learning use was positively associated with self-efficacy (*B* = 0.214, SE = 0.054, *β* = 0.212, *z* = 4.000, *p* < 0.001), supporting H1. AI literacy was also positively associated with self-efficacy (*B* = 0.211, SE = 0.060, *β* = 0.186, *z* = 3.534, *p* < 0.001). Self-efficacy was positively associated with perceived academic performance (*B* = 0.162, SE = 0.047, *β* = 0.199, *z* = 3.448, *p* < 0.001), supporting H2. The direct association between generative AI-assisted learning use and perceived academic performance did not reach the conventional 0.05 significance level (*B* = 0.090, SE = 0.046, *β* = 0.110, *z* = 1.950, *p* = 0.051, 95% CI [− 0.001, 0.181]).

**Table 4:** Baseline latent structural paths and indirect association.

Panel A. Structural associations						
Path	*B*	SE	*β*	*z*	*p*	95% Wald CI
GAIU → SE	0.214	0.054	0.212	4.000	< 0.001	[0.109, 0.319]
AIL → SE	0.211	0.060	0.186	3.534	< 0.001	[0.094, 0.328]
GAIU → PAP	0.090	0.046	0.110	1.950	0.051	[−0.001, 0.181]
SE → PAP	0.162	0.047	0.199	3.448	< 0.001	[0.070, 0.254]

Panel B. Indirect association			
Indirect association	*B*	*BootSE*	95% BC CI
GAIU → SE → PAP	0.035	0.014	[0.014, 0.068]

*N* = 376. Panel A reports maximum-likelihood estimates from the baseline latent structural model without the product-indicator interaction; the confidence intervals in Panel A are Wald intervals. Panel B reports the indirect association based on 5,000 bias-corrected bootstrap resamples. B, unstandardized estimate; *β*, standardized estimate; BC CI, bias-corrected confidence interval.

The baseline indirect association was evaluated using 5,000 bias-corrected bootstrap resamples. As shown in Table 4, Panel B, the indirect association between generative AI-assisted learning use and perceived academic performance through self-efficacy was positive (*B* = 0.035, *BootSE* = 0.014, 95% BC CI [0.014, 0.068]). Because the confidence interval excluded zero, H3 was supported as an associational hypothesis. This estimate represents a cross-sectional indirect association and does not establish temporal or causal mediation.

### 4.6 Item-level latent interaction and conditional indirect associations

The item-level latent interaction model showed good fit, χ^2^(937) = 985.674, CFI = 0.996, TLI = 0.996, RMSEA = 0.012, and SRMR = 0.034. In this final interaction model, the positive association between generative AI-assisted learning use and self-efficacy remained statistically significant (*β* = 0.207, *p* < 0.001), and self-efficacy remained positively associated with perceived academic performance (*β* = 0.199, *p* < 0.001). These coefficients are estimates from the interaction model and therefore differ slightly from the corresponding estimates in the baseline model reported in Table 4.

The latent interaction between generative AI-assisted learning use and AI literacy was positive (*B* = 0.068, *BootSE* = 0.029, *β* = 0.132, 95% BC CI [0.012, 0.123]; normal-theory *z* = 2.475, *p* = 0.013). The confidence interval excluded zero, indicating that the association between generative AI-assisted learning use and self-efficacy varied across levels of AI literacy and supporting H4.

As shown in Figure 2 and Table 5, Panel A, the association between generative AI-assisted learning use and self-efficacy was not statistically significant at low AI literacy (− 1 SD; *B* = 0.104, *BootSE* = 0.076, 95% BC CI [− 0.048, 0.249]). The association was positive at the mean of AI literacy (*B* = 0.209, *BootSE* = 0.055, 95% BC CI [0.098, 0.316]) and at high AI literacy (+1 SD; *B* = 0.314, *BootSE* = 0.065, 95% BC CI [0.186, 0.443]). Thus, the positive association was larger at higher levels of AI literacy.

**Figure 2:**
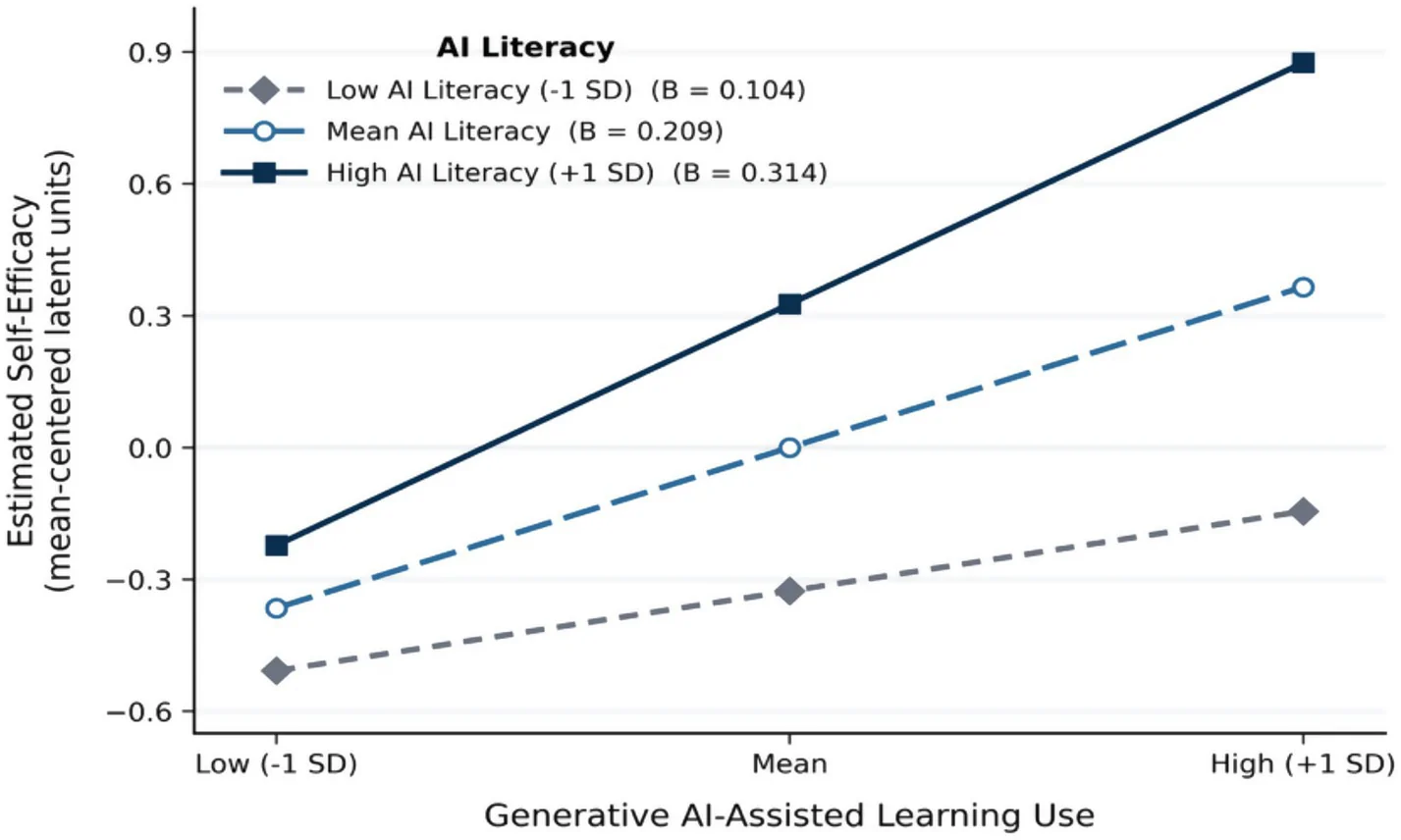
Estimated latent interaction between generative AI-assisted learning use and AI literacy in relation to self-efficacy.

**Table 5:** Latent interaction, simple slopes, and conditional indirect associations.

Panel A. Latent interaction and simple slopes predicting self-efficacy				
Effect or AIL level	*B*	*BootSE*	*β*	95% BC CI
GAIU × AIL → SE	0.068	0.029	0.132	[0.012, 0.123]
Low AIL (−1 SD)	0.104	0.076		[−0.048, 0.249]
Mean AIL	0.209	0.055		[0.098, 0.316]
High AIL (+1 SD)	0.314	0.065		[0.186, 0.443]

Panel B. Conditional indirect associations and index of moderated mediation			
AIL level or index	*B*	*BootSE*	95% BC CI
Low AIL (−1 SD)	0.017	0.013	[−0.006, 0.049]
Mean AIL	0.034	0.013	[0.014, 0.068]
High AIL (+1 SD)	0.051	0.018	[0.022, 0.094]
Index of moderated mediation	0.011	0.006	[0.002, 0.026]

Unstandardized conditional estimates are reported as B. Low and high AI literacy correspond to one standard deviation below and above the mean, respectively. Bootstrap standard errors and bias-corrected confidence intervals were based on 5,000 resamples. BC CI, bias-corrected confidence interval.

As shown in Table 5, Panel B, the conditional indirect association between generative AI-assisted learning use and perceived academic performance through self-efficacy was not statistically significant at low AI literacy (*B* = 0.017, *BootSE* = 0.013, 95% BC CI [− 0.006, 0.049]). It was positive at the mean (*B* = 0.034, *BootSE* = 0.013, 95% BC CI [0.014, 0.068]) and at high AI literacy (*B* = 0.051, *BootSE* = 0.018, 95% BC CI [0.022, 0.094]). The index of moderated mediation was positive (*B* = 0.011, *BootSE* = 0.006, 95% BC CI [0.002, 0.026]), supporting H5.

Because all focal variables were measured concurrently from the same respondents, neither the latent interaction nor the conditional indirect estimates establish temporal ordering or causality.

## 5 General discussion

### 5.1 Interpretation of the main findings

The present research examined a theory-guided conditional pattern of associations among generative AI-assisted learning use, self-efficacy, AI literacy, and perceived academic performance. The item-level latent model indicated that more frequent generative AI-assisted learning use was associated with higher self-efficacy and that higher self-efficacy was associated with more favorable perceived academic performance. The positive latent interaction indicated that the association between generative AI-assisted learning use and self-efficacy was stronger at higher levels of AI literacy. The corresponding conditional indirect association was statistically significant at the mean and high levels of AI literacy but not at the low level. These findings are consistent with the proposed theoretical framework but do not establish the temporal or causal sequence implied by that framework.

The magnitudes of the focal estimates were modest. In the baseline model, the standardized association between generative AI-assisted learning use and self-efficacy was *β* = 0.212, and the association between self-efficacy and perceived academic performance was *β* = 0.199; in the interaction model, the latent interaction was *β* = 0.132. The conditional indirect estimates ranged from 0.017 to 0.051. Statistical significance should therefore not be interpreted as evidence of a large or transformative educational association.

First, students reporting more frequent generative AI-assisted learning use also reported higher self-efficacy. This association is compatible with theoretical accounts emphasizing feedback, vicarious experience, and scaffolding (Bandura, 1997; Hattie and Timperley, 2007; Wood et al., 1976), but the present data do not show that AI-generated feedback or scaffolding caused an increase in students’ confidence. Moreover, the use measure captures frequency across heterogeneous activities and does not distinguish reflective feedback engagement from direct answer generation. The coefficient should therefore be interpreted as a modest positive association rather than evidence that more frequent AI use necessarily improves students’ capabilities.

Second, self-efficacy was positively associated with perceived academic performance and statistically accounted for part of the association between generative AI-assisted learning use and perceived academic performance. This pattern is consistent with the social cognitive proposition that capability beliefs are relevant to how students evaluate their academic functioning. However, self-efficacy and perceived academic performance are both subjective self-evaluations measured at the same time. Their association may partly reflect shared positive self-perception, reverse or reciprocal relationships, or metacognitive overconfidence. Ease or speed of task completion with AI may produce a feeling of mastery without a corresponding improvement in independently demonstrated knowledge or writing quality, a possibility consistent with research on illusions of learning and competence (Bjork et al., 2013). Self-efficacy should therefore be described as an intervening variable in an indirect statistical association, not as an empirically established causal mechanism.

Finally, AI literacy was associated with heterogeneity in the relationship between generative AI-assisted learning use and self-efficacy. The positive association was stronger among students with higher AI literacy and was not statistically distinguishable from zero at the low level of AI literacy. One possible interpretation is that students with greater AI literacy engage with AI-generated information more critically and purposefully. Nevertheless, the cross-sectional interaction does not demonstrate that AI literacy caused students to convert AI use into capability beliefs. Unmeasured differences in learning strategy, prior academic competence, digital experience, or use quality may also contribute to the observed pattern.

The Chinese platform context provides an additional qualification. Domestic and global systems may differ in accessibility, Chinese-language performance, content-policy boundaries, and the kinds of responses available to students. These differences could be associated with both reported use and students’ opportunities to exercise evaluative AI literacy. Because the questionnaire did not estimate platform-specific use or output characteristics, the present coefficients should not be interpreted as evidence that all generative AI systems operate equivalently in Chinese higher education.

### 5.2 Theoretical contributions

First, this study provides a theory-guided account of conditional covariance among generative AI-assisted learning use, self-efficacy, AI literacy, and perceived academic performance. Existing studies have examined the educational applications and implications of generative AI from different perspectives. For instance, Kasneci et al. (2023) together with Yan et al. (2024) highlighted how large language models can facilitate educational activities by supporting learning, providing formative feedback, assisting content development, and enhancing instructional practices. Similarly, Dwivedi et al. (2023) examined both the benefits and the emerging challenges associated with generative conversational AI across academic research, professional practice, and policy development. Chan (2023) proposed an AI education policy framework from the perspective of university governance. Chan and Hu (2023) revealed students’ dual perceptions of the benefits and risks of generative AI, while Cotton et al. (2024) highlighted the challenges that ChatGPT poses to academic integrity in higher education. Building on this previous research, the present study moves beyond a sole focus on AI adoption by identifying an indirect statistical association involving self-efficacy and by showing that the first-stage association varies across levels of AI literacy. This contribution concerns the organization and conditionality of the observed associations rather than the establishment of a causal psychological mechanism.

Second, the present study extends the application of social cognitive theory and self-efficacy theory to generative AI-assisted learning by positioning self-efficacy as an intervening correlate between reported AI use and perceived academic performance. The results are consistent with the proposition that students’ capability beliefs are relevant to how external learning resources are associated with learning-related perceptions. However, the present evidence does not demonstrate that technological resources were cognitively converted into learning outcomes.

Third, this study positions AI literacy as a boundary variable associated with the strength of the relationship between generative AI-assisted learning use and self-efficacy. Whereas technology-acceptance research has primarily examined adoption intentions, the present results indicate that similar levels of reported AI use may correspond to different levels of self-efficacy across students with different levels of AI literacy. This pattern does not demonstrate that AI literacy causes a more effective conversion of AI support into self-efficacy; rather, it identifies statistically significant heterogeneity in the observed association.

Fourth, the study identifies heterogeneity in the indirect association between generative AI-assisted learning use and perceived academic performance. By estimating the interaction at the item level, the latent model accounts for measurement error in the focal constructs and avoids reliance on composite-score interaction analysis. The statistically significant index of moderated mediation indicates that the indirect association varied across levels of AI literacy. The contribution therefore lies in identifying a conditional pattern of latent associations, not in proving that AI literacy causally changes a learning mechanism.

### 5.3 Practical implications

First, universities should guide students to use generative AI as a learning scaffold rather than as an answer-generation tool. Generative AI can be used for topic development, literature comprehension, outline construction, text feedback, and revision reflection. Accordingly, instructional designs should emphasize reflective use, feedback evaluation, and student-generated revision rather than assume that greater frequency of AI use itself produces educational benefits.

Second, academic writing instruction should place greater emphasis on cultivating students’ self-efficacy. Instructors may integrate generative AI into process-oriented writing instruction. For instance, students can be asked to compare multiple AI-generated outlines, use AI feedback to revise an initial draft, explain why they accepted or rejected certain AI suggestions, and reflect on differences between the original and revised versions. Such practices would allow instructors to evaluate whether engagement with AI feedback is accompanied by changes in students’ task understanding, perceived control, and actual performance rather than assuming such changes from use frequency alone.

Third, universities should incorporate AI literacy education into the core curriculum of the generative AI era. AI literacy education should go beyond tool-operation training. It should include understanding the capabilities and limitations of generative AI, learning how to formulate effective prompts, evaluating the accuracy and reliability of AI-generated content, and following academic integrity and ethical norms. Southworth et al. (2023) suggested that universities should develop AI literacy through cross-curricular design, while UNESCO (2023) emphasized that the educational use of generative AI should be grounded in human agency, transparency, and ethical responsibility.

Accordingly, all students should be supported in using AI for deeper learning, feedback integration, and critical revision, with additional scaffolding and explicit verification guidance provided to students who report lower AI literacy.

### 5.4 Study limitations and future directions

The findings reported here should be interpreted with several considerations in mind. To begin with, the evidence was obtained from a single-wave questionnaire investigation, which restricts the extent to which causal inferences can be drawn. The direction of the observed associations may be reversed or reciprocal, and the estimated indirect association should not be interpreted as evidence of a temporal mediating mechanism. Future investigations may benefit from employing longitudinal tracking or experimental approaches to explore how generative AI use, self-efficacy, and perceived academic performance evolve over time and influence one another.

Second, the dependent variable was perceived academic performance rather than an objective achievement measure. A higher score may represent accurate self-assessment, generalized positive self-evaluation, or an illusion of competence produced by easier AI-assisted task completion. The present results therefore cannot establish that students learned more, wrote better, or obtained higher grades. Future studies should combine self-reports with course grades, blinded writing assessments, instructor evaluations, delayed knowledge tests, and learning-platform behavioral records.

Third, although academic writing was an important application context in the recruitment materials and theoretical discussion, the generative AI-assisted learning use measure also covers course-content explanation, summarization, idea generation, and learning management, while self-efficacy and perceived academic performance are general academic constructs. The findings should therefore be interpreted as applying to reported generative AI-assisted learning in higher education rather than to writing-specific performance. Future research should use writing-specific measures of AI use, writing self-efficacy scales, and objective writing assessments when making claims specifically about academic writing.

Fourth, participants were recruited from Chinese universities and reported using a mixture of domestic and global generative AI systems. The survey did not measure platform-specific access, model version, content restrictions, response quality, or platform-specific use frequency. The study therefore cannot determine whether the observed associations vary across particular AI platforms or regulatory environments. Future studies should collect tool-level usage data and compare domestic and global systems directly.

Fifth, this study treated AI literacy as an overall moderator. Future research could further distinguish among dimensions such as awareness, use, evaluation, and ethics, and compare the specific roles of different AI literacy dimensions.

Sixth, this study focused primarily on the positive side of the observed association pattern involving generative AI-assisted learning. However, the use of generative AI in education may also involve risks such as overreliance, accuracy problems, ethical concerns, and assessment fairness issues. Farrokhnia et al. (2024), based on a SWOT analysis, noted that ChatGPT offers advantages such as personalized learning and efficiency improvement, while also raising potential concerns about accuracy, ethics, overreliance, and fairness in assessment. Future studies may develop dual-path models that examine how generative AI use is associated with both favorable and adverse learning processes and outcomes in higher education.

## 6 Conclusion

Overall, greater reported generative AI-assisted learning use was associated with higher self-efficacy, and higher self-efficacy was associated with more favorable perceived academic performance. AI literacy was associated with heterogeneity in the relationship between generative AI-assisted learning use and self-efficacy, and the corresponding positive indirect association was statistically significant at the mean and high levels of AI literacy but not at the low level. These findings describe a theory-consistent pattern of cross-sectional associations and should not be interpreted as evidence that generative AI use improves academic performance or that AI literacy causes the proposed psychological process. Longitudinal and experimental research is needed to establish temporal and causal ordering.

## Data Availability

The raw data supporting the conclusions of this article will be made available by the authors, without undue reservation.
