# Supplementary material for: Generative AI-assisted learning and perceived academic performance in higher education: the indirect role of self-efficacy and the moderating role of AI literacy
Source: Front Psychol. 2026 Oct 6;17:1949060. doi: 10.3389/fpsyg.2026.1949060 (PMC13680825; doi:10.3389/fpsyg.2026.1949060)
Supplement: Supplementary file 1 [file Supplementary_file_1.docx]

**APPENDIX**

Appendix A

**Table A1.** Demographic and Background Questions.

| **Variable** | **Coding** |
| --- | --- |
| Gender | 1 = Male; 2 = Female |
| Grade | 1 = Freshman; 2 = Sophomore; 3 = Junior; 4 = Senior |
| Generative AI use experience | 1 = Less than 1 month; 2 = 1–3 months; 3 = 4–6 months; 4 = 7–12 months; 5 = More than 1 year |

**Table A2.** Measurement Items.

| **Construct** | **Code** | **Measurement Item** | **Source** |
| --- | --- | --- | --- |
| Generative AI-assisted Learning Use | GAIU1 | I have used ChatGPT to get feedback on the texts I created. | Köhler & Hartig (2024) |
| Generative AI-assisted Learning Use | GAIU2 | I have used ChatGPT to have content explained to me that was not immediately clear to me in class/lecture. |  |
| Generative AI-assisted Learning Use | GAIU3 | I have used ChatGPT to create an outline for a writing project. |  |
| Generative AI-assisted Learning Use | GAIU4 | I have used ChatGPT to have emails pre-written for me. |  |
| Generative AI-assisted Learning Use | GAIU5 | I have used ChatGPT to have texts summarized. |  |
| Generative AI-assisted Learning Use | GAIU6 | I have used ChatGPT to have texts summarized. |  |
| Generative AI-assisted Learning Use | GAIU7 | I have used ChatGPT to get content ideas for a paper. |  |
| Generative AI-assisted Learning Use | GAIU8 | I have used ChatGPT to create text modules. |  |
| Generative AI-assisted Learning Use | GAIU9 | I have used ChatGPT to motivate myself for a task (e.g. using ChatGPT to talk about my stress and anxiety). |  |
| Generative AI-assisted Learning Use | GAIU10 | I have used ChatGPT to get advice (e.g. on time management). |  |
| Self-Efficacy | SE1 | Compared with other people or things, I prefer to spend time on AI chatbots. | Pintrich et al. (1991) MSLQ Self-Efficacy for Learning and Performance |
| Self-Efficacy | SE2 | Even if not actively using AI chatbots, I keep them logged in or running in the background. |  |
| Self-Efficacy | SE3 | I am spending increasingly more time on AI chatbots. |  |
| Self-Efficacy | SE4 | For me, life without AI chatbots would be inconvenient. |  |
| Self-Efficacy | SE5 | My family really tries to help me. |  |
| Self-Efficacy | SE6 | I get the emotional help and support I need from my family. |  |
| Self-Efficacy | SE7 | I can talk about my problems with my family. |  |
| Self-Efficacy | SE8 | My family is willing to help me make decisions. |  |
| AI Literacy | AIL1 | I can distinguish between smart devices and non-smart devices. | Wang, Rau, & Yuan (2023) AILS |
| AI Literacy | AIL2 | I do not know how AI technology can help me. R |  |
| AI Literacy | AIL3 | I can identify the AI technology employed in the applications and products I use. |  |
| AI Literacy | AIL4 | I can skilfully use AI applications or products to help me with my daily work. |  |
| AI Literacy | AIL5 | It is usually hard for me to learn to use a new AI application or product. R |  |
| AI Literacy | AIL6 | I can use AI applications or products to improve my work efficiency. |  |
| AI Literacy | AIL7 | I can use AI applications or products to improve my work efficiency. |  |
| AI Literacy | AIL8 | I can choose a proper solution from various solutions provided by a smart agent. |  |
| AI Literacy | AIL9 | I can choose the most appropriate AI application or product from a variety for a particular task. |  |
| AI Literacy | AIL10 | I always comply with ethical principles when using AI applications or products. |  |
| AI Literacy | AIL11 | I am never alert to privacy and information security issues when using AI applications or products. R |  |
| AI Literacy | AIL12 | I am always alert to the abuse of AI technology. |  |
| Perceived Academic Performance | PAP1 | I meet the official performance requirements expected of a student. | Verner-Filion & Vallerand (2016) |
| Perceived Academic Performance | PAP2 | I adequately complete assigned academic duties. |  |
| Perceived Academic Performance | PAP3 | I fulfill responsibilities specified in the course outline, such as studying, homework, readings, and papers. |  |
| Perceived Academic Performance | PAP4 | I perform the academic tasks that are expected of me. |  |
| Perceived Academic Performance | PAP5 | My academic performance is beyond the required demands. |  |
